# Isolation, Identification and Characterization of Two Kinds of Deep-Sea Bacterial Lipopeptides Against Foodborne Pathogens

**DOI:** 10.3389/fmicb.2022.792755

**Published:** 2022-02-03

**Authors:** Yanjun Gu, Rikuan Zheng, Chaomin Sun, Shimei Wu

**Affiliations:** ^1^College of Life Sciences, Qingdao University, Qingdao, China; ^2^CAS and Shandong Province Key Laboratory of Experimental Marine Biology, Institute of Oceanology, Chinese Academy of Sciences, Qingdao, China; ^3^Laboratory for Marine Biology and Biotechnology, Qingdao National Laboratory for Marine Science and Technology, Qingdao, China; ^4^Center of Ocean Mega-Science, Chinese Academy of Sciences, Qingdao, China

**Keywords:** *Bacillus*, deep-sea, lipopeptides, foodborne pathogens, fengycin, surfactin

## Abstract

Under multiple stresses of deep sea, many microorganisms have evolved potentials to produce different metabolites to cope with the stresses they face. In this study, we isolated a bacterial strain *Bacillus* sp. YJ17 from the deep-sea cold seep. Compared with commercial food preservative nisin, it showed broad and strong antibacterial activities against foodborne pathogens, including multiple resistant bacteria *Pseudomonas aeruginosa* PAO1 and methicillin-resistant *Staphylococcus aureus* (MRSA). The active agents were purified by reversed-phase high performance liquid chromatography (RP-HPLC). Analysis of high-energy collision induced dissociation mass spectrometry (HCD-MS) showed that the two active agents belong to family of fengycin and surfactin, and based on results of tandem mass spectrometry (HCD-MS/MS), the amino acid sequence of purified fengycin and surfactin might be Glu-Orn-Tyr-Thr-Glu-Val-Pro-Gln-Tyr-Ile and Glu-Leu/Ile-Leu/Ile-Leu/Ile-Val-Asp-Leu/Ile, respectively. Since the purified fengycin and surfactin exhibited strong inhibition against *P. aeruginosa* PAO1 and MRSA respectively, the inhibition mechanisms of fengycin against *P. aeruginosa* PAO1 and surfactin against MRSA were investigated by electron microscopy. After treatment with purified fengycin, the morphology of *P. aeruginosa* PAO1 became abnormal and aggregated together, and obvious cytoplasmic leakage was observed. After treatment with purified surfactin, the MRSA cells clustered together, and cell surface became rough and jagged. Further study showed that reactive oxygen species (ROS) accumulation and cell membrane damage occurred in *P. aeruginosa* PAO1 and MRSA after treated with fengycin and surfactin, respectively. Furthermore, typical ROS scavenging enzymes catalase (CAT) and superoxide dismutase (SOD) were also significantly reduced in *P. aeruginosa* PAO1 and MRSA after treated with fengycin and surfactin, respectively. Therefore, the inhibition mechanisms of fengycin against *P. aeruginosa* PAO1 and surfactin against MRSA are closely related with accumulation of ROS, which might be due to the decreased activity of CAT and SOD after treated with fengycin and surfactin, respectively. Overall, our study provides good candidates from the deep-sea environment to deal with foodborne pathogens, especially multidrug-resistant bacteria.

## Introduction

Under multiple stresses in the environment, microorganisms have evolved different mechanisms in order to survive, such as secreting diverse secondary metabolites to compete with other organisms for nutrients or living space (Williams et al., 1989). Members of the genus *Bacillus* have been described as potential biological control agents for their ability to produce various bioactive substances, such as lipopeptides, polyketides, and volatile metabolites (Caulier et al., 2019; Jiao et al., 2021). Lipopeptides have been widely studied because of their various biological activities, such as anti-bacterial, anti-fungal, anti-tumor and anti-virus activities (Meena and Kanwar, 2015; Zhao et al., 2017; Meena et al., 2019). According to amino acid sequences and fatty acid branches, lipopeptides can be classified into three families: iturin, surfactin, and fengycin (Romero et al., 2007; Yang et al., 2015). Iturin is composed of heptapeptide containing β-amino fatty acids (Zhou et al., 2020). Surfactin is formed by a C_12_-C_16_ β-hydroxy fatty acid linked to heptapeptide (Bonmatin et al., 1995). Fengycin is connected by a β-hydroxyl fatty acid and a decapeptide, forming a circular lactone ring like surfactin (Yang et al., 2015; Zhang and Sun, 2018).

*Listeria monocytogenes, Salmonella choleraesuis, Staphylococcus aureus* and *Pseudomonas aeruginosa* are common foodborne pathogens, which often cause serious foodborne diseases and have become a global public health problem (Zarei et al., 2014; Patra and Baek, 2017; Yasmin et al., 2017). However, the overuse of antibiotics, especially different kinds of antibiotics, has led to the increase of multidrug resistant bacteria and the accelerated spread of antibiotic resistance genes (Bjerketorp et al., 2021). Many studies have shown that lipopeptides produced by *Bacillus* have effective antibacterial activity against resistant bacterial strains (Gudina et al., 2016; Zhao et al., 2018). Due to their specific amphiphilic structure, the antibacterial mechanisms of lipopeptides are different from conventional antibiotics, mainly by destroying the integrity of microbial cell membrane or cell wall, forming holes in the membrane, allowing the leakage of cell contents and killing cells, so as to show lower drug resistance (Banat et al., 2010; Yount and Yeaman, 2013; Patel et al., 2015; Ben Ayed et al., 2017). In addition, lipopeptides have the excellent characteristics of low toxicity, biodegradability, environmental friendliness and tolerance to extreme environmental conditions (Cameotra and Makkar, 2004), which confer lipopeptides the promising potential utilization value in food health, biological prevention and medical treatment.

In this study, a marine bacterium *Bacillus* sp. YJ17 was isolated from the cold spring of the South China Sea, which exhibited broad antibacterial activity against common foodborne pathogens, and two corresponding active agents were purified. The structures of the purified active agents were analyzed by tandem mass spectrometry, and the antibacterial mechanisms were also investigated in details.

## Materials and Methods

### Stain Isolation, Culture Conditions and Strain Identification

The sediments used in this experiment were collected from the cold seep in the South China Sea (119°17′05.3940″E, 22°06′58.7264″N) at a depth of about 1,173 m in June 2020. The sediments were separated from the above samples after serial dilution with sterile seawater and incubated in 2216E agar medium (5 g tryptone, 1 g yeast paste, 15 g agar, 1,000 ml filtered seawater, pH adjusted to 7.4–7.6) at 28°C. For screening of strains with antibacterial activity, indicator bacteria were incubated in LB agar medium (10 g tryptone, 5 g yeast extract, 10 g NaCl, 15 g agar, pH adjusted to 7.0) and Trypticase Soy Broth (TSB) agar medium (17 g tryptone, 3 g Plant peptone, 5 g NaCl, 2.5g K_2_HPO_4_, 2.5 g glucose, 15 g agar, pH adjusted to 7.1–7.5) at 28°C.

For the identification of the isolated strain, the corresponding 16S ribosomal DNA (rDNA) was amplified using universal forward primer 27F (AGAGTTTGATCCTGGCTCAG) and reverse primer 1492R (TACGGCTACCTTGTTCGACTT). The high-fidelity PCR enzyme KOD One ™ was used in this process. The PCR products were mixed with 6 × loading buffer (Qingdao, Tsingke) and the bands were separated by one-percent agarose gel electrophoresis, and the corresponding fragments were recovered by the gel recovery kit and sequenced by company (Qingdao, Tsingke). The obtained sequences were compared with 16S rDNA sequences in NCBI database^1^ using the BLAST algorithm. Phylogenetic trees were constructed by MEGA-X to determine its species information.

### Screening of Strains With Antimicrobial Activity

*Pseudomonas aeruginosa* PAO1, methicillin-resistant *Staphylococcus aureus* (MRSA), *Salmonella choleraesuis* and *Listeria monocytogenes* are known to be foodborne pathogens and used as indicator strains to screen marine bacteria with high antimicrobial activity. The assay for antimicrobial activity of the isolated strains was performed as previously described (Fan et al., 2017). Briefly, to prepare the screening plate, the overnight broth culture of the foodborne pathogens was added to 50–60°C LB medium, then mixed well and poured into the plate. To screen the strain with antibacterial activity, the isolated marine strains were inoculated in screening plate, and incubated at 28°C for 48 h. If the isolated strain can secrete substances with antimicrobial activity against the indicator bacteria, an inhibition zone will be observed around the colony of the isolated strain. To further detect the antimicrobial activity of the strain with inhibition zone, the strain was inoculated into LB medium, and incubated at 28°C for 48 with a shaking speed of 150 rpm, then the supernatant was collected and the antimicrobial activity was detected against the indicator strains.

### Isolation and Purification of Antimicrobial Agents From *Bacillus* sp. YJ17

To purify the antimicrobial agents produced by *Bacillus* sp. YJ17, overnight cultures of *Bacillus* sp. YJ17 were inoculated in 250 ml conical flasks containing 100 ml LB liquid medium and incubated at 28°C for 48 h with a shaking speed of 150 rpm. The fermentation broth was centrifuged at 8,000 × *g* for 10 min at 4°C, and the cell free supernatant was precipitated by adjusting the pH to 2.5 with 6 N HCl, which was stored overnight at 4°C. The precipitate was obtained by centrifugation, then washed with 50 ml of distilled water, air-dried, and suspended in 100% methanol. The crude methanol extract was filtered through a 0.22 μm nylon membrane and injected into a reverse high performance liquid chromatography (RP-HPLC) (Agilent 1260) with an Eclipse XDB-C_18_ column (5 μm, 250 × 4.6 mm, Agilent) for further purification. Mobile phase A was water and methanol (30:70, vol/vol), and mobile phase B was 100% methanol, and elution was carried out at a flow rate of 2 ml/min under the following conditions: 0–45 min, 0% B to 100% B, then 45–60 min, 100% B. Monitoring was performed using a 210 nm UV detector, and the elution products of each peak were collected manually to detect their inhibitory activity against the indicator bacteria.

### Mass Spectrometry Analysis of Antimicrobial Agents

To obtain molecular mass information of antimicrobial agents, high-energy collisional dissociation (HCD) of active elution fractions was performed using linear ion trap Orbitrap spectrometer (LTQ Orbitrap XL; Thermo Fisher, United States), a well-established mass spectrometric cleavage technique that produces more fragments and higher quality mass spectra to enhance identification (Wang et al., 2020). The m/z values were measured from 150 to 2,000. HCD-MS-MS was used to analyze the fragment ions and further determine the structure of the antimicrobial agent. The following conditions were used for data acquisition: electrospray ion source (ESI); 3 KV spray voltage; dry gas was nitrogen, pressure was kept at 0.05 mpa; ion transfer capillary temperature was 275°C; HCD collision gas was helium, anion mode detection; collision energy was 45–60 ev. Then, Xcalibur2.1 was used to analyze the results.

### Activity Assay of Purified Antimicrobial Agents Against Foodborne Pathogens

To determine the antimicrobial activity of the purified antimicrobial agents, growth inhibition tests were performed against Gram-negative and Gram-positive foodborne pathogens according to the previously described method with some modifications (Medeot et al., 2017). Antibacterial activity assay plates were prepared as described in “Screening of Strains with Antimicrobial Activity” above. The bacteria used were: MRSA, *S. choleraesuis*, *L. monocytogenes*, *P. aeruginosa* PAO1, *Pseudomonas oryzihabitans*, *Vibrio vulnificus*, *Bacillus cereus* and *Escherichia coli*. Purified fengycin and surfactin were prepared in methanol at final concentration of 1 mg/ml, and crude methanol extract (1 mg/ml) and nisin (1 mg/ml) were prepared simultaneously. In addition, the supernatant of *Bacillus* sp. YJ17 was filtered through a 0.22 μm microporous membrane and the inhibition assay was performed. The same amount of sterile water, LB medium and methanol was used as a negative control.

### Ultrastructural and Morphological Observation of Indicator Strain After Treated With Purified Antimicrobial Agents

The effects of antimicrobial agents on foodborne pathogens *P. aeruginosa* PAO1 and MRSA were investigated by scanning electron microscopy (SEM) and transmission electron microscopy (TEM). This experiment was conducted in 24-well plates, which was added 50 μl of antimicrobial agent at a final concentration of 200 μg/ml and 1 ml LB broth inoculated with 1% overnight culture of indicator strain, then incubated in shaker at 28°C 150 rpm for 18 h. The suspension was centrifuged at 3,000 rpm for 15 min and the supernatant was aspirated off, and the precipitate was fixed by slowly adding pre-cooled 2.5% glutaraldehyde along the wall of the tube. Fixed cells were washed three times with 0.1 M phosphate buffer solution (PBS), eluted with ethanol gradient and other steps, and then observed by SEM (S-3400N; Hitachi, Tokyo, Japan) and TEM (HT7700; Hitachi, Tokyo, Japan), respectively. The same treatment was performed on the control group, with methanol instead of the antimicrobial agent. Three replicates were conducted in this assay.

### Detection of Reactive Oxygen Species Levels and Cell Integrity of Indicator Strain After Treated With Purified Antimicrobial Agents

In order to detect reactive oxygen species (ROS) levels and cell integrity of the indicator bacteria after treated with purified antimicrobial agents, when *P. aeruginosa* PAO1 and MRSA grow to the OD_600_ of 0.3, fengycin and surfactin were added to *P. aeruginosa* PAO1 and MRSA at the final concentration of 200 μg/ml, respectively, and incubated at 28°C for 4 h. After incubation, 2′,7′-dichlorofluorescein diacetate (DCFH_2_-DA; Sigma-Aldrich) dye was added and incubated for 30 min under dark conditions, then the cells were observed under a fluorescent microscope with a filter (488 nm/525 nm). Similarly, to detect the cell integrity, *P. aeruginosa* PAO1 and MRSA were treated with the same concentration of fengycin or surfactin, then stained with propidium iodide (PI) dye for 30 min under dark conditions and observed under fluorescent microscope with a 535 nm/615 nm filter. The same treatment was performed on the control group, with methanol instead of the fengycin or surfactin.

### Studies on the Intracellular Catalase and Superoxide Dismutase Activities of Indicator Strain

The effects of purified fengycin and surfactin on the activity catalase (CAT) and superoxide dismutase (SOD) in *P. aeruginosa* PAO1 and MRSA cells were assayed with corresponding assay kits (Solarbo, Beijing, China). *P. aeruginosa* PAO1 and MRSA were treated with purified fengycin or surfactin at the concentration of 0 and 200 μg/ml respectively, then incubated overnight at 28°C at a speed of 150 rpm. To obtain the total protein, the bacterial cells were collected and ultrasonicated in corresponding extraction solution, then centrifuged at 8,000 *g* for 10 min under 4°C, and the supernatant was placed on ice for testing. For CAT enzyme activity determination, 1 ml of CAT assay solution and 35 μl of supernatant were added to a 1 ml quartz cuvette and mixed for 5 s, then the absorbance was measured at 240 nm immediately, after reacted for 1 min, the absorbance was measured again, then the activity of CAT was calculated according to the absorbance at 240 nm. One CAT unit was defined as the relative degradation amount of 1 μmol of H_2_O_2_ by 1 mg of protein in one minute. For SOD activity determination, 90 μl of supernatant was added to corresponding SOD assay solution, while 90 μl distilled water was added in the control group, then incubated at 37°C for 30 min, and the absorbance was measured at 560 nm. The SOD activity was calculated according to the absorbance at 560 nm. One SOD unit was defined as enzyme activity which inhibit formation rate of blue formazan at 50% by 1 mg of protein. The absorbance was detected with a microplate reader (Infinite M200 Pro; Tecan, Switzerland).

## Results

### Screening and Identification of Antimicrobial Strains

**FIGURE 1:**
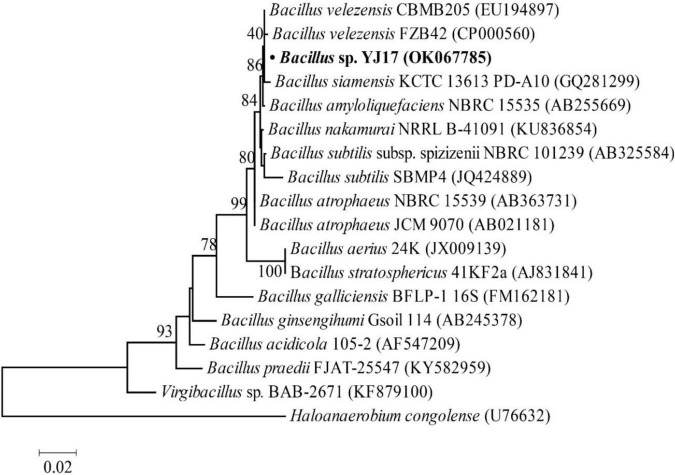
Neighbor-joining phylogenetic tree based on 16S rDNA gene sequences (1,000 bootstrap replicates).

In order to obtain strains that might produce antimicrobial agents, about 300 strains of deep-sea marine bacteria were isolated and purified from cold spring sediments in the South China Sea, and their inhibitory activities against common foodborne pathogens were detected on screening plates. For the strains with antimicrobial activities, their fermentation broth was further measured to quantify their antibacterial activity. Among them, strain YJ17 showed significant inhibitory activity against detected foodborne pathogens. As shown in Table 1, the supernatant of strain YJ17 showed inhibition circle diameters of 6.5, 13.53, 3.22, and 24.17 mm against *P. aeruginosa* PAO1, MRSA, *S. choleraesuis* and *L. monocytogenes*, respectively. The 16S rDNA sequence of strain YJ17 was sequenced and deposited into NCBI database under accession number OK067785, which exhibited high homology with *B. velezensis* strain CBMB205 (99.93%) and *B. velezensis* strain FZB42 (99.45%) in NCBI database. The phylogenetic tree was constructed by the neighbor-joining algorithm, which showed that *B. velezensis* and *B. siamensis* are the most closely related neighbors to strain YJ17 (Figure 1). Therefore, strain YJ17 was designated as *Bacillus* sp. strain YJ17.

**TABLE 1:** Inhibition spectrum of purified fengycin and surfactin.

Indicator strains	Inhibition zone (mm)				
	Supernatant (100 μl)	Methanol extract (1 mg/ml)	Fengycin (1 mg/ml)	Surfactin (1 mg/ml)	Nisin (1 mg/ml)
MRSA	13.53 ± 0.66	16.53 ± 0.43	5.56 ± 1.48	9.48 ± 0.87	–
*L. monocytogenes*	24.17 ± 1.04	17.17 ± 0.29	16.83 ± 0.28	–	–
*S. choleraesuis*	3.22 ± 0.25	2.27 ± 0.75	2.16 ± 0.25	–	–
*P. aeruginosa*	6.5 ± 0.5	5.77 ± 0.25	6.96 ± 0.50	–	–
*P. oryzihabitans*	21.33 ± 1.04	15.44 ± 1.01	16.17 ± 0.76	3.83 ± 0.76	–
*V. vulnificus*	4.66 ± 0.57	4.15 ± 0.21	2.11 ± 0.11	1.28 ± 0.26	–
*B. cereus*	12.07 ± 0.90	13.2 ± 1.04	7.06 ± 0.40	2.97 ± 0.50	–
*E. coli*	12.5 ± 1.32	5.83 ± 0.28	6.83 ± 1.04	–	–

*–, no inhibition zone.*

### Isolation and Purification of Antimicrobial Agents Produced by *Bacillus* sp. YJ17

**FIGURE 2:**
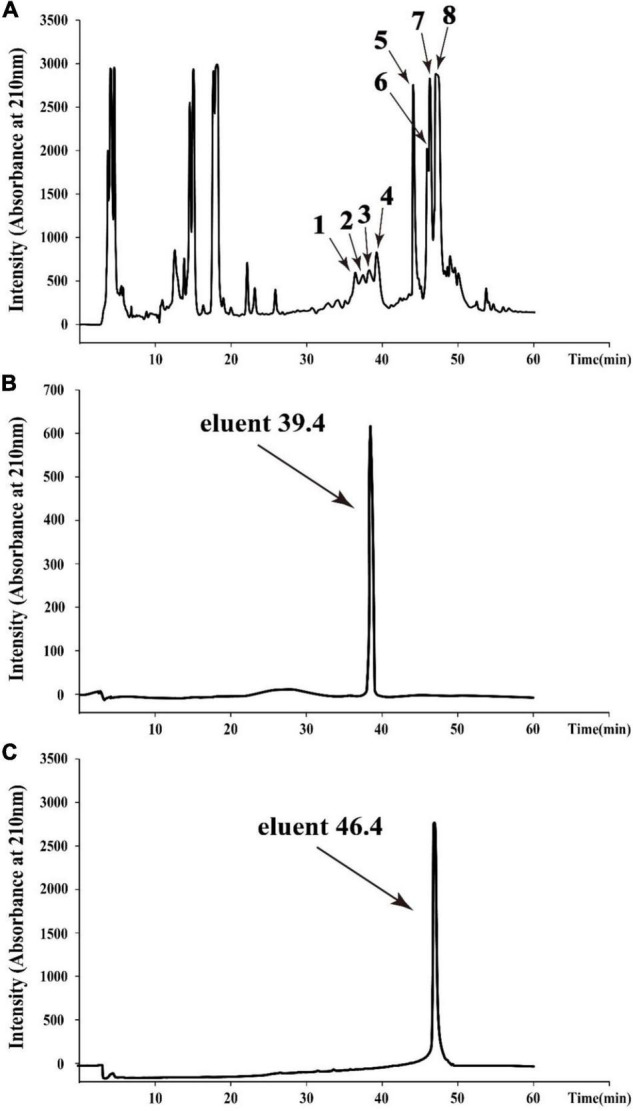
HPLC chromatogram of the antimicrobial agents produced by *Bacillus* sp. YJ17 **(A)** and further purified antimicrobial fractions of eluent 39.4 **(B)** and eluent 46.4 **(C)**.

To obtain agents with antibacterial activity from *Bacillus* sp. YJ17, the fermentation broth was purified by acid precipitation, methanol extraction and RP-HPLC. In the final purification step, eight different fractions with antibacterial activity from the crude extract were purified by RP-HPLC with the elution time at 36.6, 37.6, 38.5, 39.4, 44.2, 46.1, 46.4, and 47.2 min, respectively (Figure 2A). Among them, the strongest inhibitory activity was observed in the fractions with elution time of 39.4 min (peak 4) and elution time of 46.4 min (peak 7), which were designed as eluent 39.4 and eluent 46.4, respectively. Therefore, these two antimicrobial active fractions were further purified and analyzed in the next steps (Figures 2B,C).

### Mass Spectrometry Analysis of Purified Antimicrobial Agents

**FIGURE 3:**
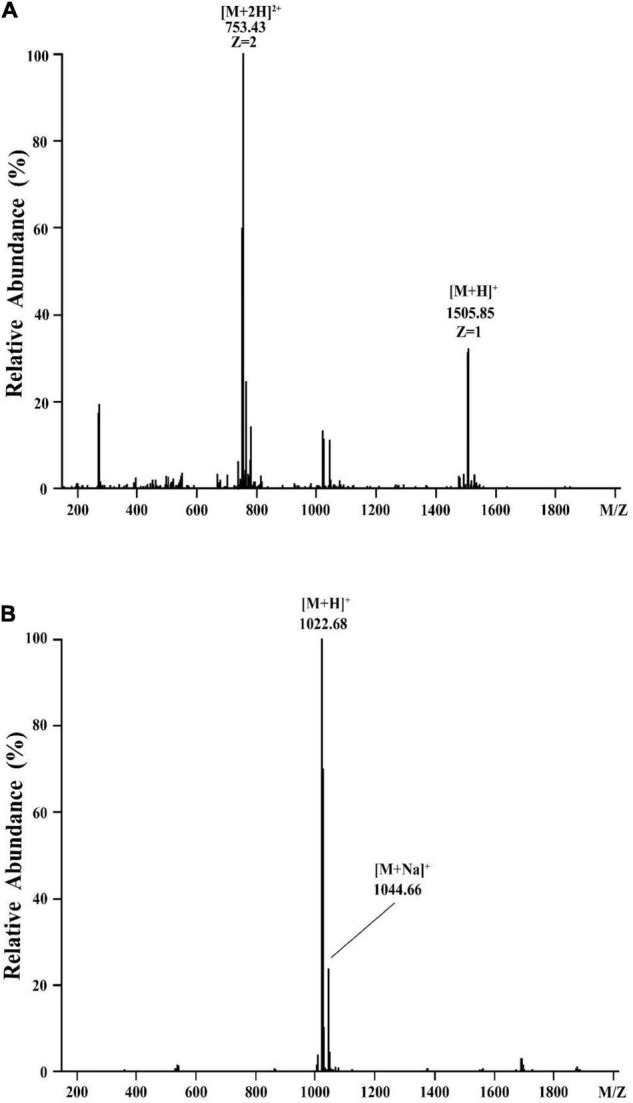
HCD-MS analysis of eluent 39.4 **(A)** and eluent 46.4 **(B)** produced by *Bacillus* sp. YJ17.

To determine the molecular mass of purified active agents, the purified fractions of eluent 39.4 and eluent 46.4 were analyzed by HCD-MS (Figure 3). For the active fraction of eluent 39.4, two peaks at m/z values of 753.43 and 1505.86 were detected, which correspond to the doubly protonated molecular ion [M + 2H] ^2+^ and the singly protonated molecular ion [M + H] ^+^, respectively. Combined with our purification procedure and previous reports on fengycin, most of them were detected at m/z values of 1463.80, 1477.81, 1491.83, 1505.84, and 1519.86 (Monaci et al., 2016), eluent 39.4 was presumed to belong to the category of fengycin. For the active fraction of eluent 46.4, a singly protonated ion [M + H] ^+^ at m/z 1022.68 and a sodium cationized ion [M + Na] ^+^ at m/z 1044.66 were detected, respectively. Based on previously reported for the surfactin, which showed typical m/z values at 999.64, 1008.65, 1022.67, 1036.68, and 1050.70 (Yang et al., 2015; Monaci et al., 2016), eluent 46.4 was presumed to belong to the category of surfactin. Therefore, the two antimicrobial agents produced by *Bacillus* sp. YJ17 belonged to lipopeptide type of fengycin and surfactin.

### HCD-MS-MS Analysis of Purified Antimicrobial Agents

**FIGURE 4:**
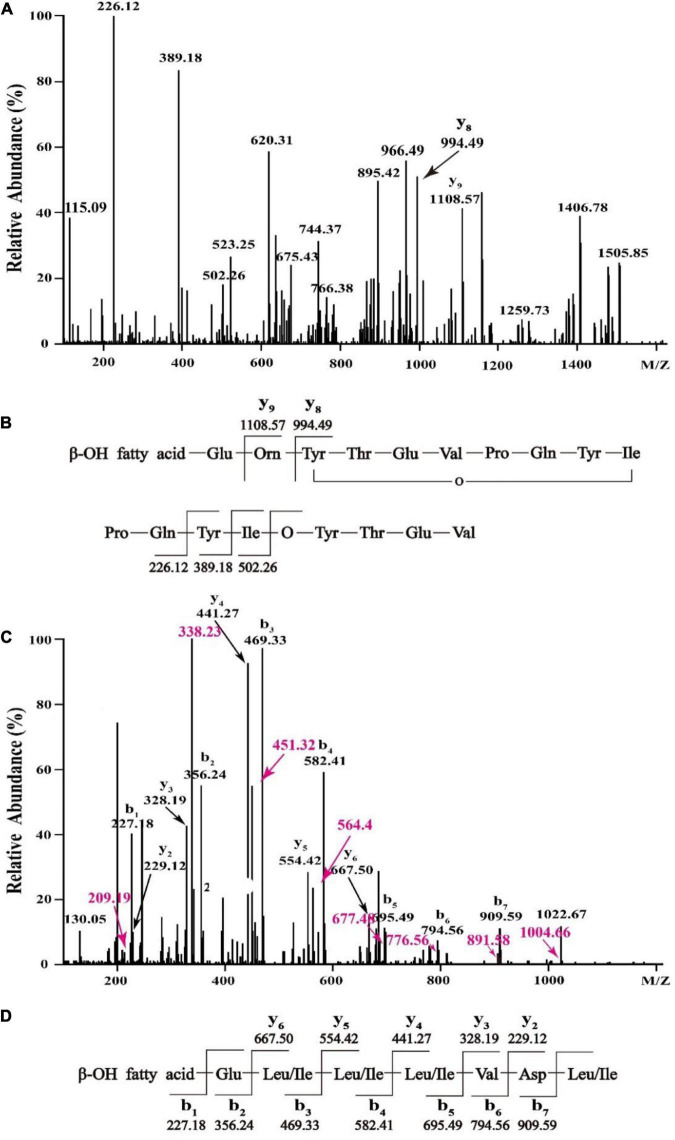
HCD-MS-MS analysis of eluent 39.4 **(A,B)** and eluent 46.4 **(C,D)** produced by *Bacillus* sp. YJ17.

In order to figure out the primary peptide sequences of eluent 39.4 and eluent 46.4, the purified fractions were further analyzed by HCD-MS-MS. For the active fraction of eluent 39.4 (Figure 4A), typical y ion fragments m/z 1108.57 and m/z 994.49 were detected, which were due to the breakage at Glu-Orn and Orn-Tyr bonds, resulting in the loss of ion fragments in the N terminal fatty acid-Glu and fatty acid-Glu-Orn. And this loss of ionic fragments usually results in the formation of specific nine peptide (Orn-Tyr-Thr-Glu-Val-Pro-Gln-Tyr-Ile) and octapeptide (Tyr-Thr-Glu-Val-Pro-Gln-Tyr-Ile). Ion fragments 226.12, 389.18, and 502.26 were also detected, which indicates the peptide sequence might be Pro-Gln-Tyr-Ile starting from the N terminus in order, which are formed by the breakage of the Val-Pro bond of a specific octapeptide ring ion. So, the amino acid sequence of fengycin might be Glu-Orn-Tyr-Thr-Glu-Val-Pro-Gln-Tyr-Ile (Figure 4B). It is noteworthy that the analytical results are consistent with previously report about C_17_-fengycin B (Liu and Sun, 2021). Therefore, it can be inferred that the eluent 39.4 is C_17_-fengycin B.

For the active fraction of eluent 46.4, the b and y ion fragments were shown in Figure 4C. The detectable b ion fragments in order from the N terminus are 909.59(b_7_), 794.56(b_6_), 695.49(b_5_), 582.41 (b_4_), 469.33 (b_3_), 356.24 (b_2_), and 227.18 (b_1_), while the given value of [M + H] ^+^ is 1022.68, and the differences between these two values in order is consistent with Leu/Ile, Asp, Val, Leu/Ile, Leu/Ile, Leu/Ile and Glu fragment ions. Starting from the C terminus, the detectable y ion fragments are in the order of 667.50 (y_6_), 554.42 (y_5_), 441.27 (y_4_), 328.19 (y_3_) and 229.12 (y_2_), and the difference between them exactly coincide with Leu/Ile, Leu/Ile, Leu/Ile, Val ion fragments. The results were identical to the analysis of b ion fragments above. Therefore, it was tentatively concluded that the primary amino acid sequence of eluent 46.4 was β-OH fatty acid-Glu-Leu/Ile-Leu/Ile-Leu/Ile-Val-Asp-Leu/Ile as shown in Figure 4D, which was different from that of previously reported surfactins (Zhao et al., 2017, 2018).

### *In vitro* Antimicrobial Activity of Purified Fengycin and Surfactin

In order to further clarify the activities of antimicrobial agents produced by *Bacillus* sp. YJ17, the supernatant, crude methanol extract and purified fengycin and surfactin were measured against common foodborne pathogens. As showed in Table 1, fengycin exhibited inhibition activity against all detected foodborne pathogens, and the most sensitive pathogen to fengycin was *L. monocytogenes*, while surfactin only showed strong antibacterial activity against MRSA. Furthermore, the purified surfactin exhibited stronger antimicrobial activity against MRSA than purified fengycin did. Combining the results of the supernatant and crude methanol extracts, fengycin and surfactin maintained the main antibacterial activity of *Bacillus* sp. YJ17. Furthermore, compared with commercial food preservative nisin, fengycin and surfactin showed unique advantages in inhibiting the growth of pathogenic bacteria with broader inhibitory spectrum.

### Ultrastructural and Morphological Changes Caused by Purified Fengycin and Surfactin

**FIGURE 5:**
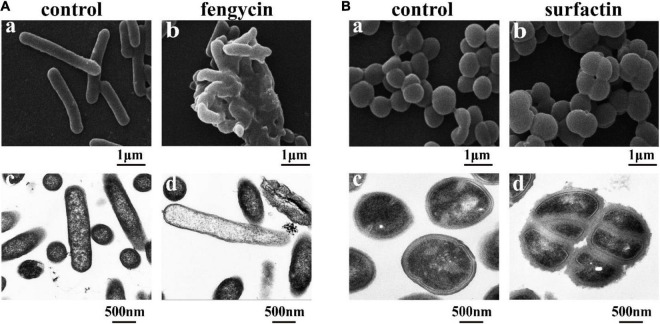
Effects of the purified fengycin and surfactin on morphology and ultrastructure of bacteria. **(A)** Effects of fengycin on the morphology and ultrastructure of *P. aeruginosa* PAO1 cells observed by SEM (a,b) and TEM (c,d). **(B)** Effects of surfactin on the morphology and ultrastructure of MRSA cells observed by SEM (a,b) and TEM (c,d). The bacterial cells in the control group were treated with the same amount of methanol, and the bacterial cells in the test group were treated with 200 μg/ml of the antimicrobial agents.

Considering that the control of infections caused by *P. aeruginosa* and MRSA is increasingly difficult due to their inherent or acquired resistance mechanisms (Kaur and Chate, 2015; Pang et al., 2019), the antimicrobial mechanisms of purified fengycin against *P. aeruginosa* PAO1 and purified surfactin against MRSA were investigated by SEM and TEM. As shown in Figure 5A, the images of *P. aeruginosa* cells without fengycin treatment showed a smooth and intact cell surface with neat edges and clear contours, and it was in uniform short rod shape without abnormal morphology under SEM (Figure 5Aa). The morphology of *P. aeruginosa* cells showed abnormal and aggregated together after being treated by fengycin (Figure 5Ab). The results from TEM showed that the *P. aeruginosa* cells in the control group had tightly bound cell walls and intact cytoplasm (Figure 5Ac). On the contrary, after being treated with fengycin, the surface of the cell wall was uneven with ruptures, and the material inside the cell was sparse, indicating that the cytoplasm was severely leaked or even all outflowed (Figure 5Ad). These results indicated that fengycin exerted its antibacterial activity against *P. aeruginosa* by causing severe damage to the cell membrane and cell wall, thus leading to cytoplasmic leakage and cell death.

Similarly, under SEM, the morphology of MRSA cells without surfactin treatment looked healthy and intact, and there was no phenomenon of multiple bacteria contracted together, while the cells clustered together after being treated with surfactin (Figures 5Ba,b). Under TEM, the MRSA cells in the control group had clear edges and uniform cytoplasm (Figure 5Bc), while the cells became rough and jagged surfaces, and increased abnormal cells clustered together after treated with surfactin (Figure 5Bd).

### Accumulation of Reactive Oxygen Species and Cell Membrane Damage of the Indicator Bacteria Caused by Fengycin and Surfactin

**FIGURE 6:**
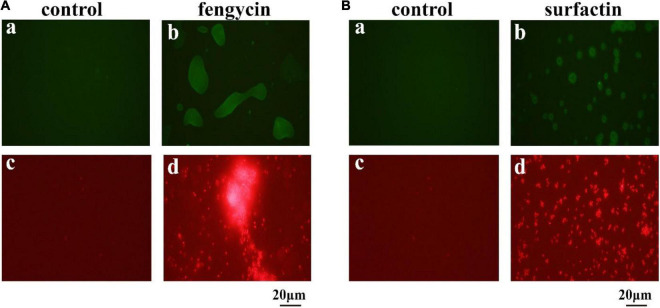
Effect of purified antimicrobial agents on ROS levels and cell integrity of *P. aeruginosa* PAO1 and MRSA under fluorescence microscopy. **(A)** Effects of fengycin on ROS accumulation (a,b) and cell membrane damage (c,d) of *P. aeruginosa* PAO1. **(B)** Effects of surfactin on ROS accumulation (a,b) and cell membrane damage (c,d) of MRSA cells. The bacterial cells in the test group were treated with the antimicrobial agents at the final concentration of 200 μg/ml, that were dissolved in methanol, and the bacterial cells in the control group were treated with the same amount of methanol.

The killing effect of several antimicrobial agents has been reported to be associated with a surge of intracellular ROS (Zhao and Drlica, 2014). Since fengycin and surfactin can cause severe inhibition to bacteria, in order to investigate whether ROS is involved in this process, ROS level was detected using the fluorescent probe DCFH_2_-DA. As shown in Figure 6, *P. aeruginosa* PAO1 and MRSA cells exhibited obvious green fluorescence after treatment with fengycin and surfactin, respectively (Figures 6Ab,Bb), while untreated control cells showed almost no green fluorescence (Figures 6Aa,Ba). The result suggests that ROS accumulation was induced in *P. aeruginosa* PAO1 and MRSA after treatment with fengycin and surfactin, respectively.

Propidium iodide can enter cells to emit red fluorescence if cell membrane is damaged and can be used as an indicator of the presence of dead cells. To further clarify whether the cell membrane of *P. aeruginosa* PAO1 and MRSA was damaged by fengycin and surfactin respectively, corresponding cells were stained by propidium iodide dye after treatment with fengycin or surfactin. As shown in Figure 6, *P. aeruginosa* PAO1 exhibited strong red fluorescence after treatment with fengycin (Figure 6Ad), and MRSA cells also showed strong red fluorescence after treatment with surfactin (Figure 6Bd), while the control group had almost no red fluorescence (Figures 6Ac,Bc). Therefore, the purified fengycin and surfactin caused the cell membrane damage of *P. aeruginosa* PAO1 and MRSA respectively, thus leading to cell death.

### Catalase and Superoxide Dismutase Activities in Indicator Bacteria

**FIGURE 7:**
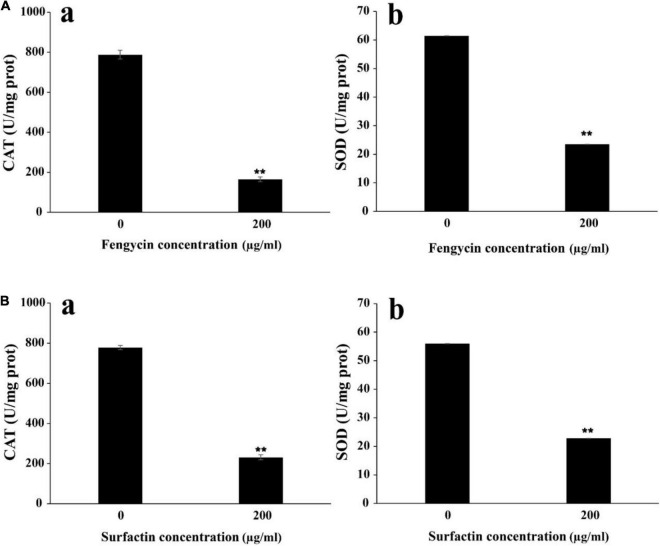
Effect of purified fengycin and surfactin on the activities of ROS scavenging enzymes CAT and SOD in *P. aeruginosa* PAO1 and MRSA. **(A)** Effect of fengycin on the activity of ROS scavenging enzymes CAT (a) and SOD (b) in *P. aeruginosa* PAO1. **(B)** Effect of surfactin on the activity of ROS scavenging enzymes CAT (a) and SOD (b) in MRSA. The error bars indicated the standard error of the mean. To denote the statistical difference between the control and treated sets, *P* values were calculated using ANOVA. **P < 0.01.

To further verify whether the ROS accumulation caused by fengycin and surfactin was related to the expression level of ROS scavenging enzymes, enzyme activities of CAT and SOD in *P. aeruginosa* PAO1 and MRSA were detected after treatment with fengycin and surfactin, respctively. The CAT activity in *P. aeruginosa* PAO1 cells was 164.80 U/mg prot after treated with 200 μg/ml of fengycin, which was far lower than that of cells treated with 0 μg/ml of fengycin (787.81 U/mg prot) (Figure 7Aa). Similarly, the CAT enzyme activity of MRSA cells was 231.09 U/mg prot after treated with 200 μg/ml of surfactin, which was also significantly reduced compared with the cells treated with 0 μg/ml of surfactin (777.93 U/mg prot) (Figure 7Ba). In addition, the SOD activities in *P. aeruginosa* PAO1 and MRSA were also strongly affected by purified fengyicn and surfactin. The SOD activity was 23.50 U/mg prot in *P. aeruginosa* PAO1 and 22.83 U/mg prot in MRSA after treatment with 200 μg/ml of fengycin and surfactin, respectively, while the SOD activities were as high as 61.44 U/mg prot and 56.02 U/mg prot when cells treated with 0 μg/ml of fengycin and surfactin (Figures 7Ab,Bb). Therefore, the activities of CAT and SOD in *P. aeruginosa* PAO1 and MRSA were dramatically reduced by fengycin and surfactin, respectively.

## Discussion

Foodborne pathogens, especially the multi-drug resistant pathogens, often cause human diseases and even lead to death (Tack et al., 2020). *Bacillus* has been generally recognized as a bio-control agent that can be safely applied in the food industry (Torres et al., 2015; Lastochkina et al., 2019), and lipopeptides produced by *Bacillus* have a well-recognized potential in controlling pathogens (Gudina et al., 2016). Normally, surfactins have been widely studied for antibacterial and their antitumor activity (Zhao et al., 2018), and the reports about fengycins have been mainly restricted to their antifungal effects (Kulimushi et al., 2017; Zhang and Sun, 2018; Jiao et al., 2021), while the effects of fengycins on bacterial cells have rarely been reported in the literature (Medeot et al., 2020).

In our study, a marine strain *Bacillus* sp. YJ17 showed antimicrobial activities against common foodborne pathogens, and three different types of lipopeptides were isolated and purified: iturin, fengycin and surfactin. Of these, iturin did not exhibit inhibitory activity against the indicator bacteria and therefore was not investigated further. Fengycin produced by this strain exhibited strong antimicrobial activity against *P. aeruginosa* PAO1, MRSA, *S. choleraesuis*, and *L. monocytogenes*, while surfactin produced by this strain showed obvious inhibition effect against MRSA. Furthermore, compared with commercial food preservative nisin, fengycin and surfactin produced by *Bacillus* sp. YJ17 showed broad and strong antibacterial activities against foodborne pathogens. Therefore, lipopeptides produced by *Bacillus* sp. YJ17 have the potential to be used as biological preservatives in the food industry and can be used as a substitute for chemical synthetic preservatives.

High concentrations of ROS have been reported to oxidize DNA, proteins and carbohydrates in organisms, leading to cell membrane damage or cell death (Rodriguez and Redman, 2005; Trachootham et al., 2009). In our study, a process accompanied by ROS accumulation and cell membrane damage was observed after the indicator bacteria were treated with fengycin or surfactin, which indicated that the ROS accumulation induced by fengycin or surfactin is an important factor leading to cell death. In addition, the activities of two typical ROS scavenging enzymes, CAT and SOD, were significantly reduced after the indicator bacteria were treated with fengycin or surfactin. Therefore, the ROS accumulation in the indicator bacteria after treated with fengycin or surfactin may due to the decreased expression of ROS scavenging enzymes, which weakens the ability to neutralize ROS and leads to the accumulation of ROS. Collectively, the cell death of indicator bacteria is closely related with the activity decrease of ROS scavenging enzymes and increase of ROS accumulation.

Altogether, both the crude extract and purified fengycin and surfactin from *Bacillus* sp. YJ17 showed strong antibacterial activity against a variety of food-borne pathogenic and spoilage bacteria, heralding their future potential in food preservative industry.

## Data Availability Statement

The original contributions presented in the study are publicly available. This data can be found here: National Center for Biotechnology Information (NCBI) database under accession number OK067785.

## Author Contributions

YG and SW conceived the study and designed the experiments. YG conducted the experiments and wrote the manuscript draft. RZ helped to conduct the experiment. CS and SW corrected the manuscript. All authors have read and approved the manuscript.

## Conflict of Interest

The authors declare that the research was conducted in the absence of any commercial or financial relationships that could be construed as a potential conflict of interest.

## Publisher’s Note

All claims expressed in this article are solely those of the authors and do not necessarily represent those of their affiliated organizations, or those of the publisher, the editors and the reviewers. Any product that may be evaluated in this article, or claim that may be made by its manufacturer, is not guaranteed or endorsed by the publisher.

## Footnotes

1
http://www.ncbi.nlm.nih.gov/BLAST/
